# Supplementary material for: Coriandrum sativum L.—Effect of Multiple Drying Techniques on Volatile and Sensory Profile
Source: Foods. 2021 Feb 12;10(2):403. doi: 10.3390/foods10020403 (PMC7918196; doi:10.3390/foods10020403)
Supplement: Supplementary file 1 [file foods-10-00403-s001.zip › Lexicon for sensory descriptive analysis.pdf]

Table 1 Cilantro descriptive sensory analysis lexicon based on Łyczko et al., (2020)

Descriptor	Definition
**Aroma**	
Anise-like	Sweet, delicate aroma resembling aniseed
Balsamic	Balsamic, spicy, slightly lemon-like odour that displays typical incense notes and is somewhat coniferous and resinous
Chamomile	Sweet, apple-like, and herbaceous
Earthy	Generally earthy, mossy, spicy, woody odour with slight phenolic and leather-like notes
Fresh	Pleasant, clean and clear aroma, with refreshing warm and sweet notes
Hay-like	Harsh, mild green, grassy floral, sweet, coumaric odour
Herbaceous	Pungent–sweet, herbal, rather musty odour with green, medicinal undertones. Reminiscent of wintergreen oil
Nuts	Nutty roasted, with slight toasted grain note
Spicy	Warm–spicy, medicinal, rather dry and almost sharp odour reminiscent of cloves;
Sweet	Sweet, very warm, floral–narcotic odour somewhat reminiscent of almonds
Vegetable	Boiled vegetables like celery, parsley and carrot roots; slightly sulphuric
Wet	Unpleasant, damp cartoon, humid basement like
Woody	Characteristic odour of toasted wood perceived by means of the sense of smell (orthonasal perception)
**Basic Tastes**	
Bitterness	Fundamental taste factor associated with a caffeine solution
Sourness	Fundamental taste factor associated with a citric acid solution
Sweetness	Fundamental taste factor associated with a sucrose solution
Saltness	Fundamental taste factor associated with a sodium chloride solution
**Flavour**	
Astringent	Puckering or drying sensation created in the mouth, throat and/or on tongue
Pungent	Sharp and pricking
Spicy	Warm–spicy, medicinal, rather dry and almost sharp, reminiscent of cloves
Aftertaste	Sensation intensity perceived just after removing the sample from the mouth or after swallowing the sample

Łyczko, J., Masztalerz, K., Lipan, L., Lech, K., Carbonell-Barrachina, Á.A., Szumny, A., 2020. Chemical determinants of dried Thai basil (O. basilicum var. thyrsiflora) aroma quality. Ind. Crop. Prod. 155, 112769. https://doi.org/10.1016/j.indcrop.2020.112769
