# Supplementary material for: Coriandrum sativum L.—Effect of Multiple Drying Techniques on Volatile and Sensory Profile
Source: Foods. 2021 Feb 12;10(2):403. doi: 10.3390/foods10020403 (PMC7918196; doi:10.3390/foods10020403)
Supplement: Supplementary file 1 [file foods-10-00403-s001.zip › Link to analyses raw datafiles.pdf]

All analyses were performed on Varian CP-3800/Saturn 2000 apparatus (Varian, Walnut Creek, CA, USA) equipped with a Zebron ZB-5MSi (30 m × 0.25 mm × 0.25 μm) column (Phenomenex, Torrance, CA, USA).. MS operational conditions were as follows: ion source temperature: 250 °C; scanning mode from 35 to 300 *m/z*.

https://drive.google.com/drive/folders/1CjkCVn1uZqmy8P--IPClRFaB3PP_gEg1?usp=sharing
