# Supplementary material for: Coriandrum sativum L.—Effect of Multiple Drying Techniques on Volatile and Sensory Profile
Source: Foods. 2021 Feb 12;10(2):403. doi: 10.3390/foods10020403 (PMC7918196; doi:10.3390/foods10020403)
Supplement: Supplementary file 1 [file foods-10-00403-s001.zip › Unknown volatile compounds mass spectra.pdf]

Both mass spectra was obtained by GC-MS analysis with Varian CP-3800/Saturn 2000 apparatus (Varian, Walnut Creek, CA, USA). MS operational conditions were as follows: ion source temperature: 250 °C; electron impact (EI) ionization at 70 eV; scanning mode from 35 to 300 *m/z*.

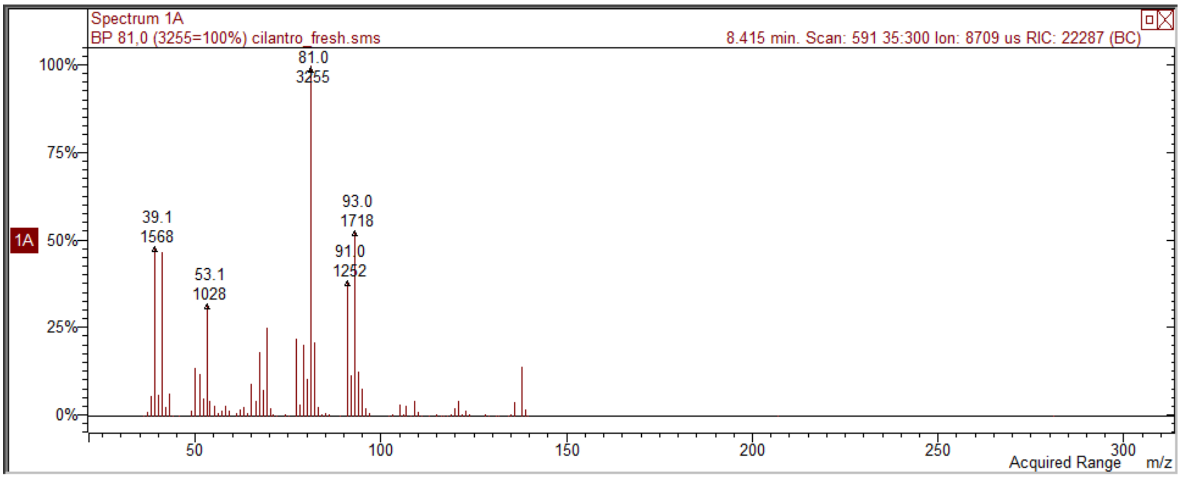

Figure S1 Unknown compound - LRI 992

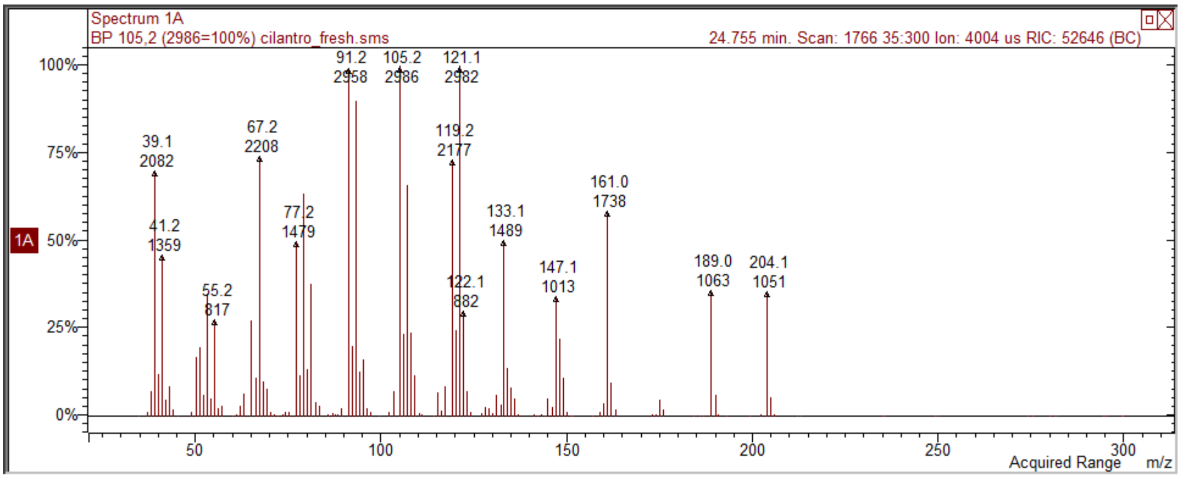

Figure S2 Unknown compound - LRI 1572
